# Human umbilical mesenchymal stem cells conditioned medium promote primary wound healing regeneration

**DOI:** 10.14202/vetworld.2016.605-610

**Published:** 2016-06-19

**Authors:** Dwi Liliek Kusindarta, Hevi Wihadmadyatami, Yuda Heru Fibrianto, Widagdo Sri Nugroho, Heru Susetya, Dewi Kania Musana, Hery Wijayanto, Surya Agus Prihatna, A. E. T. H. Wahyuni

**Affiliations:** 1 Department of Anatomy, Faculty of Veterinary Medicine, Universitas Gadjah Mada, Yogyakarta, Indonesia; 2 Department of Physiology, Faculty of Veterinary Medicine, Universitas Gadjah Mada, Yogyakarta, Indonesia; 3 Department of Veterinary Public Health, Faculty of Veterinary Medicine, Universitas Gadjah Mada, Yogyakarta, Indonesia; 4 Department of Obstetrics and Gynecology, Faculty of Veterinary Medicine, Universitas Gadjah Mada, Yogyakarta, Indonesia; 5 Department of Microbiology, Faculty of Veterinary Medicine, Universitas Gadjah Mada, Yogyakarta, Indonesia

**Keywords:** human umbilical mesenchymal stem cells conditioned medium, regenerations, wound healing

## Abstract

**Aim:**

This research was conducted to clarify the capability of human umbilical mesenchymal stem cells conditioned medium (HU-MSCM) to promote regenerations of primary wound healing on the incision skin injury.

**Materials and Methods:**

In this study, two approaches *in vitro* and *in vivo* already done. On *in vitro* analysis, tube formation was performed using HU vein endothelial cells in the presence of HU-MSCM, in some experiments cells line was incubated prior the presence of lipopolysaccharide and HU-MSCM then apoptosis assay was performed. Furthermore, *in vivo* experiments 12 female rats (*Rattus norvegicus*) were used after rats anesthetized, 7 mm wound was made by incision on the left side of the body. The wound was treated with HU-MSCM containing cream, povidone iodine was run as a control. Wound healing regenerations on the skin samples were visualized by hematoxylin-eosin staining.

**Results:**

*In vitro* models elucidate HU-MSCM may decreasing inflammation at the beginning of wound healing, promote cell migration and angiogenesis. In addition *in vivo* models show that the incision length on the skin is decreasing and more smaller, HE staining describe decreasing of inflammation phase, increasing of angiogenesis, accelerate fibroplasia, and maturation phase.

**Conclusions:**

Taken together our observation indicates that HU-MSCM could promote the acceleration of skin tissue regenerations in primary wound healing process.

## Introduction

Primary wound usually occurs as a results from operations or surgery treatments, although in this process the mortality of cellular constituents is minimal, the healing process still have some problem such as the presence of granulation tissue known as scar. The scar is one pathway of the body survival to overcome an injury as an impact from disruption of unity which may induce increasing regulation of signal from the appropriate cells and carry through the sequence of repairing without complications. Unfortunately, frequently found the ability of scar to blend in cosmetically and functionally frequently not successful and have been reported before that scar also inducible several complications such as prolonged swelling, excessive skin decortication, color or temperature changes, progressive pain, and paresthesias [1].

Nowadays, stem cells is a one of compromising technologies for therapy based on cell technology, which including as one of the parts from regenerative medicine [2]. Stem cells could be divided become several type base on its source; they are embryonic stem cells or induced pluripotent stem cells, tissue-specific stem cells, and mesenchymal stem cells (MSCs). In the last of approximately 10 years, because of the weakness from embryonic or induced pluripotent stem cells especially to the problem regarding immune-mediated rejection [3-5], thus MSCs become the major stem cells, which have been used widely for cell therapy. MSCs performed very compromising results in the treatment of numerous diseases, mainly on tissue injury and auto or alloimmune disorders [6], although some weakness still presence, e.g., in the several condition and because of culture environment MSCs can act as antigen presenting cells in addition MSCs also may conduct autoimmune conditions [7,8].

Recent discoveries suggest that microvesicles (MVs) released by MSCs during the culturing process may also important in the physiological function of the cells. Over the past few years, already known that the biological relevance of MV and nanovesicles or MSC-derived vesicles released by cells in intercellular communication, these MSC-MVs transfer proteins, lipids, and various forms of RNAs to neighboring cells known as secretom, MV, and exosome which is expected containing growth factor and cytokine, this particle mediating variety of biological responses although the physiological role of MSC-MVs is currently not well understand. However, encouraging results indicate that MSC-MVs have similar protective and reparative properties as their cellular origin in tissue repair and possibly also as anticancer therapy. Thus, MSC-MVs represent a promising opportunity to develop a novel cell-free therapy that might overcome the weakness and risks associated with the use of native MSCs [9,10].

Since only limited researches describe the advantages of MSC-MVs either on the form of secretome or exosome [11-16], it is very intriguing to describe more deeply the advantages of secretome, especially on the field of clinical trials. This present study was designed to clarify the capability of MSCs conditioned medium (MSCM) derived from umbilical cord term as human umbilical MSCM (“HU-MSCM”) as a part of secretome cell-free therapy on the basic structures of primary wound healing in the skin injury. The result is expected to support the usage of HU-MSCM as an alternative treatment.

## Materials and Methods

### Ethical approval

The use of all animal materials was approved by the Ethics Committee of the Medical Faculty, Universitas Gadjah Mada, Yogyakarta, Indonesia.

### Monoclonal antibodies (mAbs) and reagents

mAbs against αvβ3 complex (clone LM609) was purchased from Millipore, Temecula, CA, USA, recombinant human vascular endothelial growth factor (VEGF) derived from R&D, Minneapolis, MN, USA. Lipopolysaccharide (LPS) was from Sigma, Deisenhofen, Germany.

### Maintenance of HU vein endothelial cells (HUVECs)

Primary HUVECs derived from umbilical cord purchased from Lonza (Basel, Switzerland) and were cultured in endothelial cell basal medium (EBM2) (Lonza) in T-75 flask, as recommended by manufacturer. Cells were grown up to 70-80% confluence and subsequently used for the experiments. Only HUVECs maintained less than three passage was used in this study.

### Collection of HU-MSCM

When umbilical MSCs reach passage 4 in 60% confluence, cells were harvesting with trypsinization and following by centrifugation for 300 ×*g*, 10 min. Aspirate the supernatant, pellet was wash with phosphate-buffered saline (PBS) for 3 times, 10^3^ cells/ml was resuspended with complete medium (DMEM, Lonza, Basel, Switzerland), then cells would promote to become embryoid bodies and cultured on the disc. After embryoid bodies become confluence, cells were washed with PBS and complete medium without fetal bovine serum were added, after being incubated for 48 h at 37°C, 5% CO_2_. HU-MSCM was harvesting by centrifugation 300 ×*g* for 2 min.

### Detection of several cytokine in HU-MSCM

Identification of several cytokine, which included on the wound healing, was being done using the conditional medium derived from the cultivation of HU-MSCs. VEGF and fibroblast growth factor (FGF) were performed by enzyme-linked immunosorbent assay (ELISA) (R&D, Minneapolis, MN, USA), according to the manufacturer instructions. All the experiments were performed in duplicate.

### Tube formation assay

Tube formation assay through HUVECs was performed as previously described [17]. Aliquots of 50 µl ice-cold gel (Biovision, Milpitas, CA, USA) were plated onto microtiter wells (Greiner Bio-one, Frickenhausen, Germany) for 45 min at 37°C. 100 µl HUVEC (1-5 × 10^5^ cells in EBM2 medium supplemented with 2.5% fetal calf serum) was seeded carefully onto the gel for 45 min at 37°C. Thereafter, 100 µl starving EBM2 medium containing VEGF (positive control) or mAbs against αvβ3 (negative control) in final concentration 40 µg/ml or 100 µl HU-MSCM were added. Cells were allowed to grow for 16-20 h at 37°C. Data were analyzed using an F-view monochrome fluorescence microscope (Olympus, Tokyo, Japan) with 10× magnification. For the quantification of tube length, data were imported as TIFF files into ImageJ (http://imagej.nih.gov/ij/) using the stage micrometer as calibrator. All experiments were performed in duplicate.

### Apoptosis assay

Apoptosis assay was performed as previously described [17]. Cell apoptosis was measured by the Caspase-Glo3/7 assay (Promega, Madison, WI, USA). 100 µl vitronectin (2 µg/mL; Athens Research & Technology, Athens, GA, USA) was coated on 96 white well plates (Corning Incorporated, Corning, NY, USA) for 8 h. Aliquots of 250 µl HUVEC suspensions (1-2 × 10^6^ cells in EBM2 medium) were incubated with recombinant human VEGF, mAbs against αvβ3 (final concentration 20 µg/ml) and seeded onto vitronectin-coated wells for 18 h at 37°C, 5% CO_2_. In some experiment, cells were triggered by LPS 1 µg/mL for 30 min and incubated with 100 µl HU-MSCM. 100 µl of Caspase-Glo 3/7 reagents was then added at room temperature and luminescence was measured using a microtiter reader (FLX800, Biotek Instrument Winooski, VT, USA). All experiments were performed in duplicate.

### Animal model

About 12 female, white rats (*Rattus norvegicus*) with 200-300 g average of weights were derived from Integrated Testing and Research Laboratory (Lembaga Pengujian dan Penelitian Terpadu) Universitas Gadjah Mada, Yogyakarta, Indonesia. The rats were kept under standard conditions with free access of food and water 12 h daylight/dark.

#### In vivo wound healing

About 12 female, white rats (*R. norvegicus*) with 200-300 g average of weights were used in this experiments and divided become two groups. Each rat was anesthetized under administrated of 2 ml single dose ketamine (Inresa, Freiburg, Germany), and 7 mm wound was made by incision on the left side of the body. The skin wound treated topically with cream contains 1 ml HU-MSCM of mesenchymal cells in ratio 10 g cream base which containing one ml HU-MSCM medium on the wound. Povidone iodine (Meiji Indonesia, Jakarta, Indonesia) was used on as an experiment control. Both of treatments are given twice a day, for 9 days. Observation and measurement of the wounds were performed every 3 days.

During the observation days (3^rd^, 6^th^, and 9^th^ day), two rats were euthanized, and skin wound was cut and treated with Bouin’s solution (Leica, Wetzlar, Germany) continued by 70% of alcohol solution after 24 h incubations. Furthermore, each sample was processed by taking a 5 µm section and stained with hematoxylin-eosin, visualization was being done using a light microscope (Olympus, Tokyo, Japan) on the magnification of 20×.

### Statistical analysis

Statistical comparisons were made using one-way ANOVA followed by Bonferroni’s *post-hoc* test. A p<0.05 was assumed to represent statistical significance. Statistical analysis was performed with Graph Pad Prism 6 (La Jolla, CA, USA).

## Results

### HU-MSCM containing VEGF and basic FGF (bFGF)

**Figure-1:**
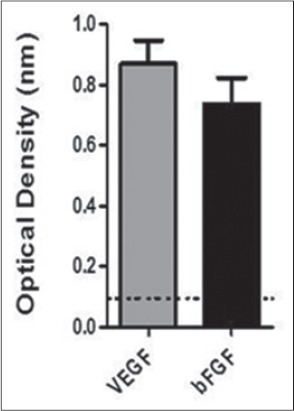
Identification of several cytokine on the human umbilical mesenchymal stem cells conditioned medium (HU-MSCM). To identified growth factor and cytokine which involved in wound healing enzyme-linked immunosorbent assay was performed, after measurement in 450 nm, could be defined HU-MSCM contain some growth factor such vascular endothelial growth factor (gray bar) and basic fibroblast growth factor (black bar).

According to our experiments using ELISA, we can describe that in HU-MSCM contained VEGF and bFGF; this evidence was conclude based on the optical density of the growth factor which is reach 0.859 and 0.736, respectively (Figure-1).

### HU-MSCM capable to decreases inflammations at the beginning of wound healing and increasing angiogenesis

**Figure-2:**
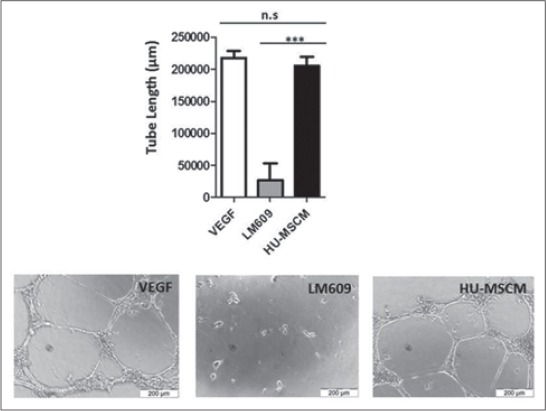
Human umbilical mesenchymal stem cells conditioned medium (HU-MSCM) promote angiogenesis. Vascular endothelial growth factor (VEGF) (white bar), monoclonal antibodies clone LM609 (gray bar), in concentration 40 µg/ml and HU-MSCM (black bar) was added to endothelial cells, and tube formation was investigated by microscopy. Data are given as mean of tube length in µm + standard deviation (upper panel). Statistical analysis was performed by one-way ANOVA followed by Bonferroni’s *post-hoc* test; n.s. = Not significant, VEGF = Control positive tube formation, LM609 = Control negative tube formation. Representative microphotographs of endothelial tube formation assays as outlined are given in the lower panel.

To investigate the possible involvement of HU-MSCM to promote angiogenesis, we performed tube formation assay, after incubation for 18 h on the matrix gel in the presence of HU-MSCM, we can describe that HU-MSCM as well as VEGF may promote angiogenesis (Figure-2) in comparison to the negative control, administrated of mAB against αvβ3 integrin (clone LM609), this antibody may inhibit adhesion of endothelial cells to the extracellular matrix lead to anoikis [18].

**Figure-3:**
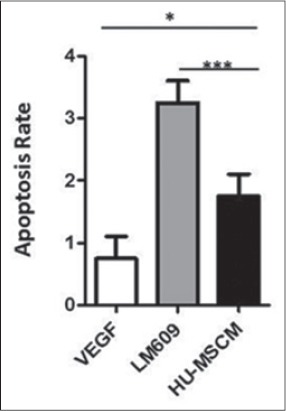
Human umbilical mesenchymal stem cells conditioned medium (HU-MSCM) could decrease apoptosis rate. Vascular endothelial growth factor (VEGF) (white bar), monoclonal antibodies (mAbs) clone LM609 (gray bar), in concentration 20 µg/ml and HU-MSCM (black bar) was added to endothelial cells and caspase 3/7 activity was measured by luminometry (upper panel). Some experiments were performed in the presence of lipopolysaccharide. Statistical analysis was performed by one-way ANOVA followed by Bonferroni’s *post-hoc* test; VEGF = Control negative for apoptosis, mAbs against αvβ3 clone LM 609 was used as positive control.

In addition, to analyze the involvement of HU-MSCM during apoptosis, caspase 3/7 assay is being done. After administrated of LPS for 45 min and following by incubation with HU-MSCM, we can conclude that HU-MSCM could decrease an apoptosis rate (Figure-3), even we can show here also there is not really true that HU-MSCM did not induce any apoptosis (Figure-3).

**Figure-4:**
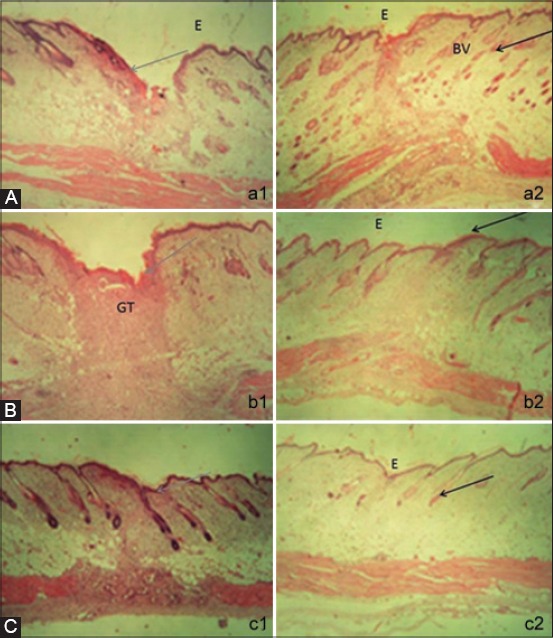
The histology of skin tissues at the 3^rd^ days of human umbilical mesenchymal stem cells conditioned medium (HU-MSCM) treatment stained with hematoxylin and eosin (H and E). (a2) Small number of inflammatory cells and abundance of blood vessels (black arrow) in compared with the control which still have abundance inflammatory cells (gray arrow) (a1) (magnification 20×) E: Epidermis, BV: Blood vessels. (b) The histology of skin tissues at the 6^th^ days of human umbilical mesenchymal stem cells conditioned medium treatment stained with hematoxylin and eosin (b2), Decreasing inflammatory cells followed by increasing re-epithelization and more density of collagen fiber (black arrow), (b1) sample from the control, a lot of inflammatory cells are found (gray arrow) (magnification 20×) E: Epidermis, GT: Granulation tissues. (c) The histology of skin tissues at the 9^th^ day of human umbilical mesenchymal stem cells conditioned medium treatment stained with hematoxylin and eosin. Fibroblasts are increasing and hair follicles created, the muscles regenerations is clearly appeared (black arrow) (c2), however, on the povidone-iodine treatment, infiltration of inflammation cells are still increasing (gray arrow) (c1) (magnification 20×). E: Epidermis.

Furthermore, on the skin tissue with the administration of cream containing HU-MSCM medium (Figure 4a2), it demonstrated that inflammation on the epidermal layer is significantly reduced, and the angiogenic process is increasing indicated by the presence of new blood vessels and reduction of neutrophil infiltrations. In contrast to the control, inflammation still gravely hamper, and there are only a few blood vessels and capillaries are appeared (Figure 4a1).

### HU-MSCM induce re-epithelialization and more density of collagen fiber

Sixth day after treatment in comparison to the control with administrations of povidone iodine (Figure 4b1), HU-MSCM medium may induce faster re-epithelialization process, reduce inflammation in granulations tissue area, and decrease the infection. HU-MSCM medium also promotes increasing density of collagen fiber (Figure 4b2).

### HU-MSCM stimulate hair follicle and muscles regenerations

On the last day of our *in vivo* observations, significantly differences between control (Figure 4c1) and treatment with HU-MSCM medium are appeared clearly. Curing of wound healing by treatment of HU-MSCM medium give compromising results indicated by stimulation of hair follicle, clearly decreased of inflammation and also significantly increasing muscle regenerations (Figure 4c2).

## Discussions

Mesenchymal conditioned medium could be defined as secreted factor alone that referred as secretome, MV, or exosome without the stem cells which may found on the medium where the stem cells are cultured. The used of MSCM as cell-free therapy have more advantages compared to the used of stem cells, especially to avoid the need to do HLA matching between donor and recipient to the important of transplant rejection, additionally MSCM more easy to produce and save in large quantity [9,19]. Our analysis regarding to the ingredient of MSCM, its can describe that MSCM may contain several cytokines such as VEGF and bFGF, our finding is in line with the recent research which some revealed in proteomics studies shows stem cells secrete various growth factors and cytokines in the conditioned medium [13,15,16]; however, during the recent years, only two clinical trial pilot study are already done to prove the advantages of MSCM [11,12].

Here, in our paper, we describe the capability of MSCM derived from HU-MSCs to promote primary curing on the incision wound on the skin. The healing of primary injury like an incision wound is a complex process which is requiring the collaborative effort of many different processes. This process including a different type of tissue and cell lineages, which contribute to proliferation, migration, matrix synthesis, inflammation, angiogenesis, maturation, also wound contractions. Growth factor and matrix signaling like developing of fibronectin, vitronectin, and integrin in general as an extracellular matrix may have also an influence for regenerations of the wound healing [20,21].

To evaluate the potency of HU-MSCM to promote wound healing, we performed some *in vitro* study. In our model, we describe that HU-MSCM may promote angiogenesis, already known during angiogenesis process besides the presence of abundantly integrin αvβ3 and avb5, VEGF together with bFGF take an important role to initiate the developing of endothelial tube formation thus angiogenesis may presence [22,23], additionally bFGF also an integral factor to activate keratinocytes, fibroblast, and endothelial cells, which are necessary presence at the beginning process of wound healing. This mechanism regarding angiogenesis are capable to push the sustainability of blood flow or blood circulation on the wound site and may help to increasing non-inflammation factor. Since the mechanism is presence, thus may guarantee for the initiation of primary wound healing and avoid the side effect of the injury.

Next, in our apoptosis assay, it revealed that HU-MSCM may decrease the apoptosis rate from the cells after the administrated of LPS, but we should realize also HU-MSCM is not really clean did not induce any apoptosis, we can speculate in here that at the early process of wound HU-MSCM capable to decrease pro-inflammatory substance which may induce to the cell death or maybe HU-MSCS may prevent the signal to mitochondria to release reactive oxygen species-mediated cell death. This mechanism is important during the early phase of healing because it will protect the new form of a blood vessel from apoptosis. In accordance to our finding, some studies have shown that the addition of MSCs to an active immune response decreases secretion of the pro-inflammatory cytokines tumor necrosis factor α and interferon-γ while simultaneously increasing the production of anti-inflammatory cytokines interleukin-10 (IL-10) and IL-4 [24-26]. In contrast, at the end of wound healing process, apoptosis signal will be increased to regarding control an excessive collagen growing thus scar could be avoided [27,28].

Furthermore, on our *in vivo* models, it shows at the beginning both of the sample (treatment and control) almost have same performance, their epidermis are in necrosis, hemorrhage on certain areas, and the presence of edema on necrotizing epidermis also infiltration of inflammation cells, but interestingly after 3^rd^ days and finally at the 9^th^ day, the structural difference between treatments and control appear significantly different. On the skin which is treated by cream containing HU-MSCM, the fibroblasts and collagen are increasing, the presence of blood vessels is visible (increasing angiogenesis), and the inflammation cells are significantly reduced, thus hair follicles are created. These results give more strongly evidence that during the wound healing process HU-MSCM may promote fibroblast proliferation resulting in intensified granulation tissue formation, the accumulation of large collagen fiber likely to increase the wound strength. HU-MSCS containing several factors which may stimulate angiogenesis, thus HU-MSCs can attract macrophages and vascular endothelial cells into the wounded tissue by releasing various angiogenic factors. In addition, an increasing expression of bFGF may partly explain the promotion of fibroblast proliferation and increased production of collagen fibers. Although accumulation of larger collagen fibers increases the wound strength, excessive accumulation of collagen may induce hypertrophic scarring, the presence of HU-MSCM will improving blood circulation on the wound, thus may increasing oxygen and also nutrient which is needed for wound curing. Reduction the amount of inflammation cells in this process may have the influence to decrease the presence of scar [29-32].

Even the HU-MSCM giving an compromising results for treatment, however, and need to be attention HU-MSCM contained cytokine as well as growth factor, and we have to realize even MSCM was derived from the same type of cells, the cells were produced by different conditions, and possible also from different passages, and culture condition, that’s why standardized methods of production, validations, and dosage of the use of MSCM need to be work out.

## Conclusions

Taking together our observations indicate that the application of HU-MSCM has a potential used as an alternative treatment for wound healing.

## Authors’ Contributions

DLK and HW designed the experiments and study; DLK, HW, WSN, HS, AETHW, DKM, HWij, SAP, and YHF performed the experiments; YHF provided essential material; DLK and HW interpreted the data and wrote the manuscript.
